# Children’s disability and caregivers’ health-related quality of life in Australia: A nationwide longitudinal study

**DOI:** 10.1007/s00431-026-07124-w

**Published:** 2026-06-23

**Authors:** Benojir Ahammed, Khorshed Alam, Zahirul Hoque, Syed Afroz Keramat

**Affiliations:** 1 School of Business, Law, Humanities and Pathways, University of Southern Queensland, Toowoomba, QLD 4350 Australia; 2 Centre for Health Research, University of Southern Queensland, Toowoomba, QLD 4350 Australia; 3 Statistics Discipline, Science, Engineering and Technology School, Khulna University, Khulna, 9208 Bangladesh; 4 School of Science, Engineering and Digital Technologies, University of Southern Queensland, Toowoomba, QLD 4350 Australia; 5 Child Health Economics Unit, Centre for Health Policy, School of Population and Global Health, The University of Melbourne, Melbourne, VIC Australia

**Keywords:** Australia, Children, Disabilities, Caregiver, Health-related quality of life

## Abstract

Childhood disability is a significant health concern with profound implications for caregivers’ well-being. This study examines the association between children’s cumulative disability profile, encompassing disability status, types, and multiplicity, and caregivers’ health-related quality of life (HRQoL) in a cross-sectional Australian setting. Utilising data from the Longitudinal Study of Australian Children (LSAC), this study examined the association between children’s cumulative disability profile and caregivers’ cross-sectional HRQoL. Caregivers’ HRQoL was assessed using the Assessment of Quality of Life-8 Dimensions (AQoL-8D) instrument. Multivariable linear regression models were estimated to assess these associations, adjusting for sociodemographic and economic covariates. Overall, 25% of children experienced at least one form of disability. A significant lower mean utility score was observed (mean difference = 0.034; 95% confidence interval [CI], [0.018, 0.050]; *p* < 0.001) among caregivers of children with disabilities compared with caregivers of children without disabilities. After adjusting for covariates, caregivers of children with disabilities had significant lower utility (*β* =  − 0.0230; 95% CI, [− 0.0376, − 0.0084]; *p* = 0.002), physical super dimension (*β* =  − 0.0339; 95% CI, [− 0.0497, − 0.0182]; *p* < 0.001), and psychological super dimension (*β* =  − 0.0181; 95% CI, [− 0.0353, − 0.0009]; *p* = 0.039) scores than caregivers of children without disabilities. Similarly, children experiencing physical, sensory, psychosocial disabilities, or multiple coexisting disabilities were associated with lower HRQoL among their caregivers compared with caregivers of children without disabilities.

*Conclusion*: Childhood disability is associated with a lower HRQoL for the caregivers.  These findings highlight the need for targeted support services, particularly for families caring for children with physical, sensory, psychosocial, and multiple disabilities, and provide estimates of reductions in health state utility scores to inform future cost-utility analyses of caregiver-focused interventions.

**Table:** 

**What is Known:** • *Caregivers of children with disabilities report lower health-related quality of life (HRQoL) and significantly higher caregiving time demands than caregivers of children without disability.* • *Despite these recognised challenges, there is a paucity of population-level evidence in the Australian context on how specific child cumulative disability profiles, including status, types, and multiple coexisting conditions, are associated with caregiver cross-sectional HRQoL.*
**What is New:** • *Using a nationally representative Australian cohort, this study identifies a significant disparity in health state utility scores between caregivers of children with disabilities and caregivers of children without disabilities.* • *Beyond overall cumulative disability status, specific categories (physical, sensory, and psychosocial), and the presence of multiple coexisting disabilities are significantly associated with lower caregivers' HRQoL across both physical and psychological dimensions.* • *The findings provide specific estimates of reductions in health state utility scores, which are essential for informing future cost-utility analyses of targeted policy interventions for Australian families.*

**Supplementary Information:**

The online version contains supplementary material available at 10.1007/s00431-026-07124-w.

## Introduction

People with disabilities represent a large global population, with around one billion individuals, or about 15% of the world’s population living with some form of disability [1]. Among them, an estimated 240 million are children [2], with 10–11% experiencing moderate to severe impairments [3]. In 2022, 12.1% of people aged 0–24 years in Australia had a disability, and over 697,100 families included at least one member with a disability [4]. Disabilities are typically classified into physical, psychosocial, sensory, and long-term health conditions, and these challenges have far-reaching consequences not only for the individuals living with them but also for their families and caregivers [1]. The consequences extend beyond the individual child, influencing the family’s emotional well-being, social relationships, and financial stability [5, 6]. Consequently, a growing number of Australian caregivers are navigating the complex and ongoing demands associated with supporting children with disabilities. Caregivers are responsible for meeting their child’s daily needs, often dedicating extensive time to caregiving activities [7], and they frequently report a wide range of adverse effects associated with these responsibilities [8].

Childhood disabilities vary in severity, complexity, and support needs, resulting in diverse disability profiles. These conditions shape children’s developmental, social, and health trajectories while also creating substantial emotional, social, and financial challenges for families, particularly primary caregivers who manage intensive care needs and coordinate multiple services [9]. Studies conducted in Australia showed that caregivers of children with disabilities commonly experience role strain, financial stress, reduced workforce participation, and fewer opportunities for health-promoting activities, which can negatively affect their physical and psychological well-being [6, 10]. Social support, employment, flexible work arrangements, and access to disability services have been identified as key protective factors for caregivers' well-being [11]. In the post National Disability Insurance Scheme (NDIS) context, many caregivers continue to face emotional and administrative challenges related to coordinating services and navigating support systems [12]. However, caregiving experiences are not solely negative, as many caregivers also report personal growth, resilience, strengthened family relationships, and a sense of meaning and purpose from caring for a child with disability [13]. Recognising both the challenges and positive aspects of caregiving is important for a balanced understanding of caregivers' health and well-being.

Health-related quality of life (HRQoL) provides a focused measure of the perceived physical, mental, and social consequences of health over time [14]. Growing recognition of the broader consequences of childhood disability has prompted increasing attention to how a child’s health state affects the HRQoL of caregivers, and to the importance of incorporating these effects into economic evaluations [15]. Globally, in some regions, the rising toll of lead-attributable intellectual developmental disorders among children and adolescents underscores the enduring challenge of childhood disability [16] and its potential implications for caregivers' HRQoL [1]. Current recommendations emphasise including caregivers' health outcomes whenever an intervention is expected to influence caregivers' well-being [17]. Evidence consistently shows that caregivers of children with disabilities report lower HRQoL due to the cumulative demands of caregiving and associated psychosocial strain [18]. However, most studies have examined single disability categories rather than diverse or co-occurring disability profiles [19–21]. This leaves a critical gap in understanding how combinations of functional impairments shape caregivers' health  outcomes. Recognising how disability status, type, and number influence caregiver HRQoL is crucial for designing targeted supports that effectively enhance caregivers' well-being [22, 23].

In Australia, where the prevalence and recognition of childhood disability are rising, and support systems such as the NDIS continue to evolve, understanding how different disability profiles are associated with caregivers' well-being is both timely and necessary. Despite growing policy emphasis on family-centred approaches and caregiver support, empirical evidence on how children’s cumulative disability characteristics vary and influence caregiver HRQoL remains limited.

The aim of this study is to examine the association between cumulative childhood disability profiles and the cross-sectional HRQoL of Australian caregivers, thereby addressing a key gap in the existing literature. By identifying the disability types and specific combinations that are associated with caregivers' health outcomes, the findings will   inform service planning, policy expansion, and economic evaluations that increasingly recognise caregivers' well-being as a critical component of child and family health.

## Materials and methods

### Data source and participants

The Longitudinal Study of Australian Children (LSAC) dataset was used for this study. LSAC comprises biannual data collected from wave 1 (2004) through wave 10 (2023) across two cohorts: the B cohort and the K cohort. The study’s design and recruitment procedures have been described in detail elsewhere [24]. A one-off physical health and biomarker module, the Child Health CheckPoint, was introduced for the B cohort between waves 6 and 7 as a cross-sectional component. Families who completed the wave 6 home interview were eligible to participate. In 2015–2016, the B cohort child (aged 11–12 years) and parent 1 attended either an extended clinic assessment or a shorter home visit, although the B cohort included 5107 children at wave 1 and 3764 at wave 6 (approximately 74% of the wave 1 sample). Only 1874 (approximately 50% of those in wave 6) participated in the Child Health CheckPoint module, which was opt-in, limited to families involved across waves 1–6, and required intensive long-time in-person physical assessments rather than standard surveys, likely reducing participation due to logistical constraints. Analyses in this study include information from both children and their caregivers. Parent 1, identified by LSAC as the individual most knowledgeable about the child, was designated as the caregiver of the respondent. As the key explanatory variable, children’s disability status at any time between waves 1 and 6 was included. Caregivers of children with disabilities often experience ongoing psychological distress and reduced HRQoL due to sustained caregiving demands [25, 26]. Longitudinal evidence further indicates that these adverse effects can persist even as the child’s condition changes, reflecting a sustained association with caregivers' well-being [27]. Caregivers' HRQoL and covariate information were taken from the Child Health CheckPoint data when the children were aged 11–12 years. Finally, a total of 1823 participants, representing more than 97% of the Child Health CheckPoint sample, were included in the final analysis. A detailed description is provided in Figure S1 (Supplementary Material).

### Outcome variable

The primary outcome variable of this study is the caregiver’s HRQoL, measured using the Assessment of Quality of Life-8 Dimensions (AQoL-8D). The AQoL-8D is a preference-based instrument widely used to assess HRQoL, which covers the domains of independent living, senses, pain, happiness, mental health, self-worth, coping, and relationships. It comprises two broad super dimensions: physical and psychological [28]. The physical super dimension (PSD) reflects functional ability and the burden of physical symptoms, as measured across domains such as independent living, senses, and pain. The psychological super dimension (PsySD) captures emotional, social, and mental well-being through domains including happiness, mental health, self-worth, coping, and relationships [29]. Together, these dimensions provide a comprehensive assessment of overall HRQoL through utility scores ranging from 0 to 1, with higher scores indicating better quality of life. The preference-based structure of the AQoL-8D also enables its use in economic evaluations [29].

### Key exposure variables

Disability status, disability type, and number of disabilities were the main exposures. Seventeen medical conditions or disabilities were identified in the dataset. The cumulative disability of children was classified dichotomously as having at least one disability or none from wave 1 to wave 6. These conditions were grouped into four broad categories: physical, sensory, psychosocial, and other disabilities or long-term conditions (LTCs) following established and recognised national and international frameworks [30–32]. Definition and detailed descriptions of these categories were presented in Table S1 (Supplementary Material). Additionally, the number of disabilities was categorised as (i) no, (ii) single, and (iii) multiple, allowing assessment of the cumulative challenges of multiple co-occurring disabilities.

### Covariates

Based on previous research and data availability, a comprehensive set of sociodemographic and economic factors was incorporated [19, 33–35]. In this study, the covariates included child sex (male, female), child age (in years), child covered by a health care card (no, yes), region of residence (major city, rest of the state), SEIFA disadvantage quintile (least disadvantaged, 2nd least, middle, 2nd most, most disadvantaged), caregiver's age (in years), caregiver's sex (male, female), caregiver's body mass index (BMI) (thinness, normal, overweight, obese), and caregiver's partner status (no, yes).

### Ethical approval

Ethical approval for the LSAC was obtained from the Australian Institute of Family Studies Human Research Ethics Committee. The study was conducted in accordance with the National Statement on Ethical Conduct in Human Research and consistent with the principles of the Declaration of Helsinki.

### Statistical analysis

Several statistical techniques were employed to analyse the data. Descriptive statistics summarised all variables, with means and standard deviations (SD) reported for continuous variables, and frequencies and percentages for categorical variables. Prior to regression analysis, the assumptions of normality and absence of multicollinearity were examined, with no major violations identified that would influence the interpretation of results. Independent samples *t*-tests were conducted to compare caregivers’ HRQoL utility scores and the two AQoL‑8D super dimensions, namely PSD and PsySD, between children with and without disabilities. Associations between children’s cumulative disability profiles and caregivers' cross-sectional HRQoL were then assessed using multivariable linear regression models. Regression coefficients (*β*) and their 95% confidence intervals (CIs) are reported. The use of multivariable regression enabled adjustment for all covariates, yielding more robust, internally valid estimates in this cross-sectional context. This analytical approach provides rigorous and reliable estimates of the association between changes in children’s disability status and caregivers’ HRQoL.

For the sensitivity analysis, missing data were addressed using a random forest–based imputation method, after which multivariable linear regression models were re-estimated. This approach is regarded as highly robust when the proportion of missing data is below 5% (Supplementary Material, Table S2). The random forest–based imputation method is a non-parametric iterative technique that predicts missing values by capturing complex, non-linear relationships among variables [36]; performs well with mixed data types; and generally yields more accurate imputations compared with simpler methods such as mean substitution or simple regression imputation [37]. Consistent with the literature, complete-case analysis generally yields unbiased or minimally biased estimates when missingness is small, even where the missing at random assumption is not strictly satisfied [38, 39].

Additionally, multivariable regression analyses were performed, with disability status from the immediately preceding survey wave (wave 6) as the primary explanatory variable, and covariates and outcome measures drawn from the Child Health CheckPoint data collected between waves 6 and 7. All analyses were conducted in RStudio (version 2024.12.0) using R (version 2025.05.1 + 513), with two-sided tests applied throughout and a statistical significance threshold set at *p* < 0.05.

## Results

### Descriptive statistics

A descriptive summary of the study participants is presented in Table 1. A total of 1823 children were included, comprising 927 male (50.9%) and 896 female (49.1%). Most children were not covered by a health care card (72.6%, *n* = 1324), and the majority resided in major cities (70.4%, *n* = 1284). Regarding socioeconomic status, 34.7% (*n* = 632) were classified in the least disadvantaged SEIFA quintile, while 8.3% (*n* = 152) were in the most disadvantaged quintile. Caregivers had a mean age of 43.7 years (SD = 5.23), and most lived with a partner (88.3%, *n* = 1609). According to caregiver's BMI, 37.8% (*n* = 690) were classified as normal weight, 32.5% (*n* = 592) as overweight, and 29.1% (*n* = 531) as obese; a small proportion, 0.5% (*n* = 10), were classified as underweight. The baseline characteristics of participants from LSAC wave 1 (*n* = 5107), comparing those included in the analytic sample (*n* = 1823) with those excluded (*n* = 3284), are presented in Table S3 (Supplementary Material).

**Table 1:** Background characteristics of the LSAC analytic samples (*n* = 1823)

Factors	Overall, *n* (%)/mean (SD)
Children characteristics	
Sex of children	
Male	927 (50.9%)
Female	896 (49.1%)
Age of children, mean (SD)	11.5 (0.5)
Child covered by a health care card	
No	1324 (72.6%)
Yes	499 (27.4%)
Region of residence	
Major city	1284 (70.4%)
Rest of state	539 (29.6%)
SEIFA disadvantage quintile	
Least disadvantaged	632 (34.7%)
2nd least	427 (23.4%)
Middle	344 (18.9%)
2nd most	268 (14.7%)
Most disadvantaged	152 (8.3%)
Caregiver’s characteristics	
Age of caregivers, mean (SD)	43.7 (5.2)
Body mass index of caregivers	
Thinness	10 (0.5%)
Normal	690 (37.8%)
Overweight	592 (32.5%)
Obese	531 (29.1%)
Partner of caregivers	
No	214 (11.7%)
Yes	1609 (88.3%)
Sex of caregivers	
Male	223 (12.2%)
Female	1600 (87.8%)

**Note: Abbreviations.** *SD*, standard deviation

**Fig. 1:**
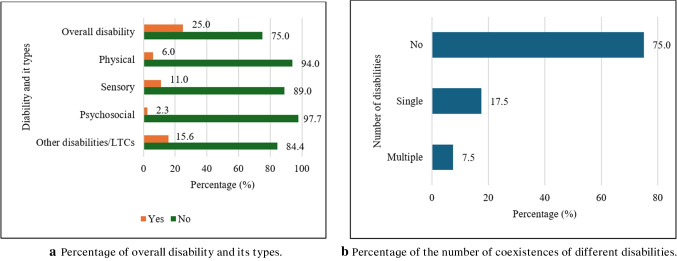
Disability profiles of the study children

Figure 1 presents the distribution of children’s disability profiles. Figure 1a shows that one in four children (25.0%) experienced at least one disability during childhood, while 75.0% experienced none. The figure also shows that 6.0% of children reported physical disabilities, 11.0% sensory disabilities, and 2.3% psychosocial disabilities. Additionally, 15.6% of children had ‘other disabilities/LTCs’. Furthermore, Fig. 1b shows that 17.5% of children had a single disability, while 7.5% experienced multiple disabilities.

**Fig. 2:**
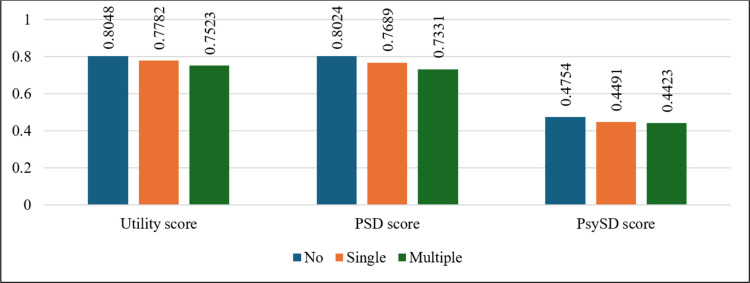
Mean HRQoL of caregivers by the number of disabilities of the study children

Fig. 2 shows the mean caregivers' health state utility score and its super dimensions (PSD and PsySD) by the number of child disabilities. A clear downward trend is evident, with caregivers' HRQoL declining as the number and complexity of the child’s disability increased. Caregivers of children without disabilities had the highest mean utility score (0.8048), followed by caregivers of children with a single disability (0.7782), while the lowest scores were observed among caregivers of children with multiple disabilities (0.7523). A similar pattern appears for PSD scores, which declined from 0.8024 (no disability) to 0.7689 (single disability) and 0.7331 (multiple disabilities). PsySD scores were also highest for caregivers of children without disabilities (0.4754) and lowest for caregivers of children with multiple disabilities (0.4423). Across all HRQoL dimensions, the presence of multiple disabilities was consistently associated with the greatest decline in caregivers' well-being.

### Comparison of HRQoL

Caregivers of children with any disability consistently and significantly reported lower HRQoL scores across all dimensions compared with caregivers of children without disabilities (Table 2). There was a significant mean difference (MD) in utility score of 0.034 (95% confidence interval [CI], [0.018, 0.050]; *p* < 0.001). Similarly, significant mean differences were observed for the PSD (MD = 0.044; 95% CI, [0.026, 0.062]; *p* < 0.001) and PsySD (MD = 0.028; 95% CI, [0.011, 0.046]; *p* = 0.002), indicating that caregivers of children with disabilities had significantly lower HRQoL than caregivers of children without disabilities.

**Table 2:** HRQoL in caregivers of children with and without disability/disability types (*n* = 1823)

HRQoL	**Disability status**				**Mean difference (95% CI)**	*p*-value
	**No**		**Yes**			
	***n***	**Mean (SD)**	***n***	**Mean (SD)**		
Utility score	1367	0.805 (0.137)	456	0.770 (0.156)	0.034 (0.018, 0.050)	< 0.001
PSD score	1367	0.802 (0.146)	456	0.758 (0.173)	0.044 (0.026, 0.062)	< 0.001
PsySD score	1367	0.475 (0.163)	456	0.447 (0.169)	0.028 (0.011, 0.046)	0.002
	**Physical disability**				**Mean difference (95% CI)**	
	**No**		**Yes**			
	***n***	**Mean (SD)**	***n***	**Mean (SD)**		
Utility score	1713	0.801 (0.139)	110	0.725 (0.175)	0.076 (0.042, 0.110)	< 0.001
PSD score	1713	0.796 (0.150)	110	0.714 (0.192)	0.083 (0.046, 0.120)	< 0.001
PsySD score	1713	0.472 (0.162)	110	0.413 (0.190)	0.059 (0.022, 0.096)	0.002
	**Sensory disability**				**Mean difference (95% CI)**	
	**No**		**Yes**			
	***n***	**Mean (SD)**	***n***	**Mean (SD)**		
Utility score	1622	0.800 (0.140)	201	0.763 (0.162)	0.037 (0.014, 0.061)	0.002
PSD score	1622	0.796 (0.149)	201	0.750 (0.184)	0.046 (0.019, 0.073)	0.001
PsySD score	1622	0.472 (0.164)	201	0.440 (0.165)	0.032 (0.007, 0.056)	0.011
	**Psychosocial disability**				**Mean difference (95% CI)**	
	**No**		**Yes**			
	***n***	**Mean (SD)**	***n***	**Mean (SD)**		
Utility score	1781	0.798 (0.140)	42	0.706 (0.204)	0.093 (0.029, 0.157)	0.005
PSD score	1781	0.793 (0.152)	42	0.707 (0.224)	0.087 (0.017, 0.157)	0.017
PsySD score	1781	0.470 (0.164)	42	0.397 (0.192)	0.073 (0.013, 0.133)	0.018
	**Other disabilities/LTCs**				**Mean difference (95% CI)**	
	**No**		**Yes**			
	***n***	**Mean (SD)**	***n***	**Mean (SD)**		
Utility score	1539	0.799 (0.140)	284	0.780 (0.158)	0.019 (0.000, 0.039)	0.055
PSD score	1539	0.797 (0.149)	284	0.758 (0.175)	0.039 (0.017, 0.061)	< 0.001
PsySD score	1539	0.469 (0.163)	284	0.463 (0.173)	0.007 (− 0.015, 0.029)	0.539

**Abbreviations:** *HRQoL*, health-related quality of life; *PSD*, physical super dimension; *PsySD*, psychological super dimension; *SD*, Standard deviation

Caregivers' HRQoL varies across four disability categories. Significant differences were observed for physical, sensory, and psychosocial disabilities across all HRQoL domains. For example, compared with caregivers of children without physical disabilities, caregivers of children with physical disabilities showed significant mean differences in utility (MD = 0.076; 95% CI, [0.042, 0.110]; *p* < 0.001), PSD (MD = 0.083; 95% CI, [0.046, 0.120]; *p* < 0.001), and PsySD (MD = 0.059; 95% CI, [0.022, 0.096]; *p* = 0.002) scores. A similar pattern was observed for sensory and psychosocial disabilities. In contrast, the ‘other disabilities/LTCs’ category showed a smaller association. Although a significant difference was observed only for PSD score (MD = 0.039; 95% CI, [0.017, 0.061]; *p* < 0.001), no meaningful differences were found in overall utility or PsySD scores of HRQoL.

### Regression results

Table 3 presents the adjusted associations between children’s cumulative disability profiles and caregivers’ cross-sectional HRQoL. Childhood disability was consistently associated with lower caregiver HRQoL. Caregivers of children with disabilities had significantly lower health state utility (*β* =  − 0.0230; 95% CI, [− 0.0376, − 0.0084]; *p* = 0.002), PSD (*β* =  − 0.0339; 95% CI, [− 0.0497, − 0.0182]; *p* < 0.001), and PsySD (*β* =  − 0.0181; 95% CI, [− 0.0353, − 0.0009]; *p* = 0.039) scores, than caregivers of children without disabilities.

**Table 3:** Association of children’s cumulative disability status, disability types, and number of disabilities (waves 1–6) with caregivers’ cross-sectional health-related quality of life (Child Health CheckPoint data between waves 6 and 7) (*n* = 1823)

	**Risk factor**	**Model 1: utility score**		**Model 2: PSD score**		**Model 3: PsySD score**	
		***β* (95% CI)**	***p*-value**	***β* (95% CI)**	***p*-value**	***β* (95% CI)**	***p*-value**
Set 1	**Disability**						
	No	ref		ref		ref	
	Yes	− 0.0230 (− 0.0376, − 0.0084)	0.002	− 0.0339 (− 0.0497, − 0.0182)	< 0.001	− 0.0181 (− 0.0353, − 0.0009)	0.039
Set 2	**Types of disability**						
	**Physical disability**						
	No	ref		ref		ref	
	Yes	− 0.0592 (− 0.0869, − 0.0314)	< 0.001	− 0.0537 (− 0.0837, − 0.0237)	< 0.001	− 0.0494 (− 0.0822, − 0.0167)	0.003
	**Sensory disability**						
	No	ref		ref		ref	
	Yes	− 0.0228 (− 0.0434, − 0.0023)	0.029	− 0.0290 (− 0.0513, − 0.0068)	0.011	− 0.0226 (− 0.0469, 0.0016)	0.067
	**Psychosocial disability**						
	No	ref		ref		ref	
	Yes	− 0.0458 (− 0.0894, − 0.0022)	0.039	− 0.0328 (− 0.0799, 0.0142)	0.172	− 0.0355 (− 0.0869, 0.0159)	0.176
	**Other disabilities/LTCs**						
	No	ref		ref		ref	
	Yes	0.0140 (− 0.0047, 0.0327)	0.142	− 0.0085 (− 0.0287, 0.0117)	0.411	0.0224 (0.0003, 0.0444)	0.047
Set 3	**Number of disabilities**						
	No	ref		ref		ref	
	Single	− 0.0179 (− 0.0338, − 0.0005)	0.035	− 0.0267 (− 0.0447, − 0.0087)	0.004	− 0.0176 (− 0.0373, 0.0019)	0.077
	Multiple	− 0.0352 (− 0.0593, − 0.0111)	0.006	− 0.0512 (− 0.0774, − 0.0250)	< 0.001	− 0.0190 (− 0.0476, 0.0096)	0.193

***Note:*** Analysis of models in sets 1, 2, and 3 adjusted for sex of children, age of children, child covered by a health care card, region of residence, SEIFA disadvantage quintile, age of caregivers, sex of caregivers, body mass index of caregivers, and partner of caregivers; *β*, regression coefficient. ***Abbreviations:**** PSD*, physical super dimension; *PsySD*, psychological super dimension; *SD*, standard deviation; *ref*, reference category; *CI*, confidence interval

The association varied by disability category. Children with physical disabilities were significantly associated with poorer caregiver HRQoL, reflected in lower health state utility (*β* =  − 0.0592; 95% CI, [− 0.0869, − 0.0314]; *p* < 0.001), PSD (*β* =  − 0.0537; 95% CI, [− 0.0837, − 0.0237]; *p* < 0.001) and PsySD (*β* =  − 0.0494; 95% CI, [− 0.0822, − 0.0167]; *p* = 0.003) scores. Similarly, children with sensory disabilities were significantly associated with lower caregiver HRQoL, as indicated by reduced utility scores (*β* =  − 0.0228; 95% CI, [− 0.0434, − 0.0023]; *p* = 0.029) and PSD scores (*β* =  − 0.0290; 95% CI, [− 0.0513, − 0.0068]; *p* = 0.011), compared with caregivers of children without sensory disabilities. Psychosocial disability in children was significantly associated with lower health state utility scores (*β* =  − 0.0458; 95% CI, [− 0.0894, − 0.0022]; *p* = 0.039). In contrast, ‘other disabilities/LTCs’ showed no meaningful associations with any of the outcomes.

The number of disabilities in children was also significantly associated with caregivers' HRQoL. Caregivers of children with a single disability had lower utility (*β* =  − 0.0179; 95% CI, [− 0.0338, − 0.0005]; *p* = 0.035) and PSD (*β* =  − 0.0267; 95% CI, [− 0.0447, − 0.0087]; *p* = 0.004) scores than caregivers of children without disabilities. The largest differences were observed among caregivers of children with multiple disabilities, who had lower utility scores (*β* =  − 0.0352; 95% CI, [− 0.0593, − 0.0111]; *p* = 0.006) and PSD scores (*β* =  − 0.0512; 95% CI, [− 0.0774, − 0.0250]; *p* < 0.001) than caregivers of children without disabilities.

Regression estimates remained consistent between children’s disability and caregivers’ HRQoL after imputing missing data (Table 4). Compared with caregivers of children without disabilities, caregivers of children with any disability were significantly associated with lower scores across all HRQoL dimensions, including utility (*β* =  − 0.0227; 95% CI, [− 0.0370, − 0.0083]; *p* = 0.002), PSD (*β* =  − 0.0335; 95% CI, [− 0.0490, − 0.0179]; *p* < 0.001), and PsySD (*β* =  − 0.0182; 95% CI, [− 0.0351, − 0.0014]; *p* = 0.034) scores.

**Table 4:** Association of children’s cumulative disability status, disability types, and number of disabilities (waves 1–6) with caregivers’ cross-sectional health-related quality of life (Child Health CheckPoint data between waves 6 and 7): analysis based on missing imputed data (*n* = 1874)

	**Risk factor**	**Model 1: utility score**		**Model 2: PSD score**		**Model 3: PsySD score**	
		***β* (95% CI)**	***p*-value**	***β* (95% CI)**	***p*-value**	***β* (95% CI)**	***p*-value**
Set 1	**Disability**						
	No	ref		ref		ref	
	Yes	− 0.0227 (− 0.0370, − 0.0083)	0.002	− 0.0335 (− 0.0490, − 0.0179)	< 0.001	− 0.0182 (− 0.0351, − 0.0014)	0.034
Set 2	**Types of disability**						
	**Physical disability**						
	No	ref		ref		ref	
	Yes	− 0.0613 (− 0.0883, − 0.0343)	< 0.001	− 0.0553 (− 0.0847, − 0.0260)	< 0.001	− 0.0529 (− 0.0847, − 0.0211)	0.001
	**Sensory disability**						
	No	ref		ref		ref	
	Yes	− 0.0217 (− 0.0420, − 0.0014)	0.036	− 0.0284 (− 0.0504, − 0.0063)	0.012	− 0.0220 (− 0.0459, 0.0019)	0.071
	**Psychosocial disability**						
	No	ref		ref		ref	
	Yes	− 0.0451 (− 0.0886, − 0.0016)	0.042	− 0.0312 (− 0.0785, 0.0160)	0.195	− 0.0348 (− 0.0860, 0.0164)	0.183
	**Other disabilities/LTCs**						
	No	ref		ref		ref	
	Yes	0.0150 (− 0.0033, 0.0334)	0.108	− 0.0066 (− 0.0266, 0.0133)	0.513	0.0230 (0.0014, 0.0446)	0.037
Set 3	**Number of disabilities**						
	No	ref		ref		ref	
	Single	− 0.0183 (− 0.0347, − 0.0019)	0.028	− 0.0270 (− 0.0448, − 0.0093)	0.003	− 0.0181 (− 0.0373, 0.0012)	0.066
	Multiple	− 0.0332 (− 0.0572, − 0.0093)	0.007	− 0.0489 (− 0.0749, − 0.0230)	< 0.001	− 0.0187 (− 0.0468, 0.0094)	0.192

***Note:*** Analysis of models in sets 1, 2, and 3 adjusted for sex of children, age of children, child covered by a health care card, region of residence, SEIFA disadvantage quintile, age of caregivers, sex of caregivers, body mass index of caregivers, and partner of caregivers; *β*, regression coefficient. ***Abbreviations:**** PSD*, physical super dimension; *PsySD*, psychological super dimension; *SD*, standard deviation; *ref*, reference category; *CI*, confidence interval

Patterns varied by disability type. Caregivers of children with physical disabilities were significantly associated with lower utility (*β* =  − 0.0613; 95% CI, [− 0.0883, − 0.0343]; *p* < 0.001), PSD (*β* =  − 0.0553; 95% CI, [− 0.0847, − 0.0260]; *p* < 0.001) and PsySD (*β* =  − 0.0529; 95% CI, [− 0.0847, − 0.0211]; *p* = 0.001) scores compared with caregivers of children without physical disabilities. Similarly, caregivers of children with sensory disabilities had lower utility (*β* =  − 0.0217; 95% CI, [− 0.0420, − 0.0014]; *p* = 0.036) and PSD (*β* =  − 0.0284; 95% CI, [− 0.0504, − 0.0063]; *p* = 0.012) scores of HRQoL, compared with their counterparts. Psychosocial disability was also significantly related to lower utility scores (*β* =  − 0.0451; 95% CI, [− 0.0886, − 0.0016]; *p* = 0.042), whereas its associations with PSD and PsySD scores were not statistically significant at the 5% level.

The number of disabilities also mattered. Caregivers of children with a single disability were significantly associated with lower utility (*β* =  − 0.0183; 95% CI, [− 0.0347, − 0.0019]; *p* = 0.028) and PSD (*β* =  − 0.0270; 95% CI, [− 0.0448, − 0.0093]; *p* = 0.003) scores, relative to caregivers of children without disabilities. Notably, the strongest associations were observed among caregivers of children with multiple disabilities, who had significantly lower utility (*β* =  − 0.0332; 95% CI, [− 0.0572, − 0.0093]; *p* = 0.007) and PSD (*β* =  − 0.0489; 95% CI, [− 0.0749, − 0.0230]; *p* < 0.001) scores compared with caregivers of children without disabilities. No statistically significant differences were found in PsySD scores between the two groups.

The regression results also showed mixed patterns across children’s immediate disability profiles and caregivers' HRQoL dimensions (Supplementary Material, Table S4). Having a child with any disability was significantly associated with lower caregiver HRQoL outcomes, with lower utility (*β* =  − 0.0334; 95% CI, [− 0.0601, − 0.0067]; *p* = 0.014), and PsySD (*β* =  − 0.0338; 95% CI, [− 0.0652, − 0.0024]; *p* = 0.035) scores. Patterns varied by disability types. Psychosocial disability in children showed the strongest association, with lower health state utility (*β* =  − 0.0912; 95% CI, [− 0.1637, − 0.0185]; *p* = 0.014). Physical and sensory disabilities had negative coefficients across all models, but these were not statistically significant at the 5% level. Children with single disabilities were associated with modest, non-significant differences in utility (*β* =  − 0.0274; *p* = 0.084) and PsySD (*β* =  − 0.0340; *p* = 0.068) scores. Multiple disabilities showed larger point estimates for lower utility (*β* =  − 0.0491; *p* = 0.052) and PSD (*β* =  − 0.0498; *p* = 0.068), although the confidence intervals included zero, indicating uncertainty. Overall, the findings indicate stronger associations for psychosocial disability and for caregivers of children with multiple conditions.

## Discussions

### Key findings

This study contributes to the literature by employing multivariable regression analysis to quantify the association between children’s cumulative disability profiles and caregivers’ HRQoL using a nationally representative Australian sample. The model’s methodological strength stems from its ability to move beyond simple associations and provide more rigorous evidence. It estimates the association between a child’s disability status, including the presence of multiple disabilities, and HRQoL, while adjusting for potential confounding factors.

The study found that one in four children is experiencing specific types of disability. Caregivers of children with disabilities reported poorer HRQoL than caregivers of children without disabilities. The decline was consistent across utility, PSD, and PsySD scores, and was most evident among caregivers of children with multiple disabilities. The main findings indicate that children’s disability was associated with significant reductions in caregivers' HRQoL. Among children’s disabilities, physical, sensory, and psychosocial conditions showed the most consistent associations with poorer caregiver well-being, although physical and sensory disabilities were more strongly related to the caregiver’s physical outcomes of HRQoL. Strongest associations were observed for physical and psychosocial disability, and for children with multiple disabilities. Overall, these findings underscore the significant association of children’s cumulative disabilities with caregivers’ HRQoL, especially when disabilities are multiple or involve physical and psychosocial conditions.

The findings of this study are consistent with earlier research showing that families of children with disabilities report a lower level of HRQoL and its super dimensions (PSD and PsySD) [1]. Several factors may explain these patterns. Childhood disability is often associated with poorer child health [40], which in turn is linked to poorer caregivers' health [41] and reduced caregivers' HRQoL [42]. Caring for a child with a disability typically involves ongoing physical and emotional demands, including frequent health appointments, behavioural challenges, and specialised medical or rehabilitative needs, all of which have been consistently associated with poorer caregivers' physical and mental health [42, 43]. These caregiving responsibilities trigger a broader cascade of psychosocial and financial consequences that erode caregivers' HRQoL. Elevated psychological stress, disrupted sleep, and chronic fatigue progressively deplete caregivers’ physical and emotional reserves, while restricting opportunities for personal care and social participation and further reducing well-being [44, 45]. Many caregivers also reduce working hours or leave employment to meet heightened costs of therapies and specialised services, compounding financial strain and further diminishing well-being [46].

Caregivers of children with physical disabilities reported poorer HRQoL, particularly in the physical and psychological dimensions, consistent with the findings from earlier studies [27, 47]. Children’s physical disabilities often require intensive and ongoing care, including lifting, mobility support, assistance with daily activities, and regular specialist visits, all of which increase caregivers’ physical workload and fatigue and are strongly associated with poorer physical and mental health outcomes [48]. These caregivers often face a greater number of medical appointments, increased time away from work, and more intensive care coordination responsibilities, which increases time pressure and limits employment opportunities [49, 50]. The additional direct and indirect costs of therapies, equipment, transport, and lost earnings associated with a child’s physical disability place significant economic pressure on the family, further lowering caregivers’ HRQoL [46].

Children often experience sensory impairments, which are associated with lower caregivers' HRQoL. This study provides clear evidence that sensory disability is associated with a significant decline in caregivers’ HRQoL. Several qualitative studies reported that caring for a child with visual impairment is associated with poorer caregivers' quality of life [51, 52]. Similarly, another study found that parents of preschool children with severe hearing impairment or speech and language disorders had significantly lower HRQoL than their counterparts, parents of children who did not have impairments [53]. Sensory disabilities often require additional family support, assistive devices, and specialised therapies, creating financial and logistical pressures such as frequent medical appointments and extensive travel. These demands place considerable strain on caregivers and reduce their overall life satisfaction, with numerous studies showing that disability-related responsibilities steadily erode caregivers’ quality of life [52, 54].

Children with psychosocial disabilities are associated with elevated caregivers' stress and reduced well-being, consistent with evidence linking behavioural and emotional difficulties in children to these outcomes [55]. Families of children with mental health conditions also report substantial emotional and financial difficulty, which is further associated with lower caregivers' HRQoL [56, 57]. Similar evidence from studies on ADHD and other psychosocial difficulties confirms increased caregivers' strain and poorer family quality of life [58].

Children with single or multiple disabilities were more likely associated with  low HRQoL of caregivers. As the number of disabilities increases, so too do the emotional, financial, and time demands placed on caregivers, resulting in poorer well-being. Families caring for children with multiple conditions experience higher levels of stress and greater caregiving responsibilities [59], and the increased need for appointments, therapies, and supervision is further associated with caregivers' fatigue and lowered HRQoL [60].

Ensuring the rights of children with multiple and complex disabilities requires healthy and well-supported caregivers on whom they can rely. Maintaining caregivers' capacity is crucial not only for the child but also for broader societal well-being, as physically and mentally well caregivers are better equipped to meet their caregiving responsibilities than those experiencing fatigue and burnout. Evidence indicates that children with disabilities face an increased risk of abuse and neglect, potentially attributable to caregiver burnout and parental mental health difficulties [61, 62]. These findings highlight that supporting caregivers' health and well-being is fundamental to ensuring the safety, health, and overall outcomes of children with disabilities.

### Magnitude and practical relevance of clinical and economic findings

When evaluating the magnitude of utility differences, it is essential to consider established thresholds of clinical significance. In health economics, the minimally clinically important difference (MCID) for preference-based measures is usually estimated between 0.03 and 0.05 [63], with changes within or above this range generally regarded as meaningful at the population level [64]. However, the MCID is not fixed; it varies depending on context, such as condition, caregiver relationship, and baseline status, and should be informed by condition-specific, validated evidence [65]. For overall cumulative disability status, the adjusted utility score was − 0.0230 (95% CI, [− 0.0376, − 0.0084]), which falls slightly below the lower limit of the MCID range with confidence intervals also spanning this threshold, indicating a modest but potentially meaningful difference at the population level. Physical, psychosocial, and multiple disabilities showed utility differences of − 0.0592, − 0.0458, and − 0.0352, respectively, which reached or surpassed the commonly cited threshold [63] and aligned with clinically meaningful differences in caregivers' HRQoL. Sensory disability indicated a smaller difference (− 0.0228), reflecting modest yet consistent variation. Nonetheless, in most estimates, overall disability status and specific disability types for the PSD and PsySD domains of HRQoL met the MCID threshold.

From a clinical perspective, differences of 0.03 to 0.05 or more in utility are generally regarded as meaningful, although thresholds vary on the context and population [63, 64, 66]. In this study, the observed differences for physical disability, psychosocial disability in some models, and the multiple disabilities often meet or surpass the 0.05 benchmark, indicating clinically meaningful differences. Reductions in the PSD reflect changes in caregivers’ physical functioning, while declines in the PsySD signify emotional strain. These findings support the incorporation of caregivers' well-being assessments into routine child disability services.

Economically, a lower level of caregivers' well-being has been linked to productivity loss, increased health care use, and broader societal costs [67, 68]. Because utility scores directly convert into quality-adjusted life years, even modest and sustained drops in caregivers' HRQoL can lead to significant economic consequences, especially when viewed across affected families at the population level [68]. There is growing awareness that health interventions create spillover effects on family members’ health, and that omitting caregivers' health state utilities from economic evaluations may underestimate the full value of interventions [15]. These findings support including caregivers' HRQoL in economic assessments of childhood disability services and pushing for targeted policy actions to enhance caregiver outcomes.

### Policy implications

In the Australian context, targeted, evidence-based policies are essential to improve caregivers' HRQoL for families of children with disabilities. First, expanding access to early intervention and treatment services through the NDIS is critical, particularly for families managing multiple disabilities, as this can reduce financial strain and lengthy wait times. Increasing the availability of short-term accommodation and bolstering carer respite programs would help alleviate caregivers' burnout and support mental health. Care coordination challenges could be mitigated by better integrating disability assistance with primary health care and school-based programs, particularly for families dealing with physical and sensory disabilities. Income support measures, such as the Carer Allowance and Carer Payment, should be reviewed to ensure they adequately reflect the additional out-of-pocket expenses associated with therapies, transportation, and assistive equipment. Workplace flexibility programs, funded by federal and state governments, are needed to enable caregivers, particularly women, to maintain employment while managing intensive care responsibilities. Finally, from a policy perspective, the study’s findings can inform future cost-utility analyses by providing robust utility estimates for children with different disabilities, thereby supporting evidence-based priority setting and resource allocation in health and social care systems. Collectively, these measures have the potential to substantially enhance caregivers' HRQoL across Australia.

### Strengths and limitations

This study offers several notable strengths. It provides new insights into how children’s disabilities, whether single, specific types, or coexisting, are associated with caregivers' HRQoL in Australia. By disaggregating disability into physical, sensory, psychosocial, and other categories and examining the cumulative influence of multiple disabilities, the analysis offers a level of granularity often absent in prior research. The use of the AQoL-8D utility score and its two super dimensions strengthens the assessment by capturing the broader physical, emotional, and psychosocial domains relevant to caregivers. Furthermore, the use of large, nationally representative data enhances the generalisability of findings across diverse family and disability contexts.

This study recognises several limitations, which point to opportunities for future research. First, reliance on a single Child Health CheckPoint wave restricts HRQoL measurement to a cross-sectional design, limiting the ability to capture changes over time or establish causal pathways, while child disability was assessed across follow-up waves. Second, the study does not account for important child and caregiver-related social, health and behavioural factors such as family structure, child BMI, severity of disability, caregiver education, caregiver mental health history, physical activity, family history of disability, or chronic health conditions, which may act as explanatory factors or confounders in the relationship between children’s disability and caregivers’ HRQoL [57, 69, 70]. These factors could intensify the observed association between childhood disabilities and caregivers' HRQoL but were not explored in this study. Third, the relatively small number of children with psychosocial disabilities may have limited the precision of the estimated associations. Fourth, although seventeen disability categories were included, several developmental and neurological conditions were not captured due to data limitations, and these may also influence caregiver HRQoL. Finally, HRQoL was assessed using only the AQoL-8D utility score to measure HRQoL; other validated instruments, such as the SF-36 [71] and the Quality of Well-Being Scale [72], could not be incorporated because they were not available in the dataset.

## Conclusions

The global rise of children’s disabilities underscores the importance of understanding its association with caregivers' well-being. This study indicates that children’s disability status, including physical and psychosocial conditions, and multiple coexisting disabilities, is strongly associated with lower caregivers' HRQoL. These findings highlight the urgent need for policy reforms to reduce the challenges associated with childhood disability for families and to improve caregivers' HRQoL. Strengthening support systems and implementing targeted interventions that address the diverse needs of children with disabilities are essential steps that align closely with the priorities of Australia’s Disability Strategy. In the Australian context, disability policies, particularly under the NDIS, should more explicitly recognise and support caregivers' health alongside child-focused outcomes. Targeted and integrated supports for families of children with physical, psychosocial, and multiple disabilities, including respite care, mental health services, and flexible employment and income support, are essential to reducing caregivers' demands and responsibilities. Incorporating caregivers' HRQoL into economic evaluations would further support evidence-based investment in family-centred disability services.

## Supplementary Information

Below is the link to the electronic supplementary material.

ESM 1 (DOCX 85.0 KB)

ESM 2 (PDF 222 KB)

## Data Availability

The data used in this study are from the Longitudinal Study of Australian Children (LSAC), which is conducted by the Australian Government Department of Social Services (DSS) and managed in partnership with the Australian Institute of Family Studies (AIFS). Access to LSAC data is subject to approval and is available to eligible researchers through the National Centre for Longitudinal Data (NCLD). More information on data access can be found at (https://dataverse.ada.edu.au/dataverse.xhtml?alias=lsac).
