# Supplementary material for: Children’s disability and caregivers’ health-related quality of life in Australia: A nationwide longitudinal study
Source: Eur J Pediatr. 2026 Jun 23;185(7):519. doi: 10.1007/s00431-026-07124-w (PMC13290929; doi:10.1007/s00431-026-07124-w)
Supplement: Supplementary file 1 — (DOCX 85.0 KB) [file 431_2026_7124_MOESM1_ESM.docx]

# **Children’s disability and caregivers’ health-related quality of life in Australia:  a nationwide longitudinal study**

**Supplementary Material**

**Tables**

**Table S1.** Definition of different types of disability.

| **Disability type** | **No.** | **Description** | **Response** | **Definition** |
| --- | --- | --- | --- | --- |
| **Physical** | 1 | Limited use of arms or fingers | No/Yes | A “yes” response to at least one of the six questions was classified as a physical disability; otherwise, it was not. |
|  | 2 | Difficulty gripping things | No/Yes |  |
|  | 3 | Limited use of legs and feet | No/Yes |  |
|  | 4 | Any condition that restricts physical work (For example, back problems, migraines) | No/Yes |  |
|  | 5 | Physical disfigurement or deformity | No/Yes |  |
|  | 6 | Has chronic or recurring pain or discomfort, causing restriction | No/Yes |  |
| **Sensory** | 1 | Has sight problems not corrected by glasses or contact lenses | No/Yes | A “yes” response to at least one of the three questions was classified as a sensory disability; otherwise, it was not. |
|  | 2 | Has hearing problems | No/Yes |  |
|  | 3 | Has speech problems | No/Yes |  |
| **Psychosocial** | 1 | Has a nervous or emotional condition causing restriction | No/Yes | A “yes” response to at least one of the two questions was classified as a psychosocial disability; otherwise, it was not. |
|  | 2 | Has a mental illness for which help or supervision is required | No/Yes |  |
| **Other disabilities/ long-term conditions (LTCs)** | 1 | Has shortness of breath or breathing difficulties, causing restriction | No/Yes | A “yes” response to at least one of the six questions was classified as an other disabilities/LTCs; otherwise, it was not. |
|  | 2 | Has blackouts, fits or loss of consciousness | No/Yes |  |
|  | 3 | Has difficulty learning or understanding things | No/Yes |  |
|  | 4 | Has a long-term condition or ailment that is still restrictive even though it is being treated or medication is being taken for it | No/Yes |  |
|  | 5 | Has other long-term conditions such as arthritis, asthma, heart disease, Alzheimer's disease, dementia etc | No/Yes |  |
|  | 6 | Long-term effects as a result of a head injury, stroke or other brain damage | No/Yes |  |

**Table S2.** Summary of missing observations.

| **Variable** | **Total observation** | **Missing observation** | **Percent Missing** |
| --- | --- | --- | --- |
| **Outcome variables** |  |  |  |
| HRQoL, AOQL-9D utility score | 1874 | 13 | 0.7% |
| PSD score | 1874 | 11 | 0.6% |
| PsySD score | 1874 | 13 | 0.7% |
| **Covariates** |  |  |  |
| Sex of children | 1874 | 0 | 0% |
| Age of children | 1874 | 0 | 0% |
| Child covered by a health care card | 1874 | 19 | 1.0% |
| Region of Residence | 1874 | 0 | 0% |
| SEIFA disadvantage quintile | 1874 | 0 | 0% |
| Age of caregivers | 1874 | 0 | 0% |
| Sex of caregivers | 1874 | 0 | 0% |
| Body mass index of caregivers | 1874 | 14 | 0.7% |
| Partner of caregivers | 1874 | 10 | 0.5% |
| **Key explanatory variable(s)** |  |  |  |
| Disability status | 1874 | 14 | 0.7% |
| Physical disability | 1874 | 14 | 0.7% |
| Sensory disability | 1874 | 14 | 0.7% |
| Psychosocial disability | 1874 | 14 | 0.7% |
| Other disabilities/LTCs | 1874 | 14 | 0.7% |
| Number of disabilities | 1874 | 14 | 0.7% |

***Abbreviations:*** HRQoL, Health related Quality of Life ; PSD, Physical super dimension; PsySD, Psychological super dimension.

**Table S3.** Baseline characteristics of LSAC Wave 1 participants (n=5107), comparing included (n=1823) and excluded participants (n = 3284).

|  | **Final analytic sample** | **Not included in the**  **final analytic sample** | **Overall** |
| --- | --- | --- | --- |
| **Factors** | **(N=1823)** | **(N=3284)** | **(N=5107)** |
| **Children characteristics** |  |  |  |
| **Sex of children** |  |  |  |
| Male | 927 (50.9%) | 1681 (51.2%) | 2608 (51.1%) |
| Female | 896 (49.1%) | 1603 (48.8%) | 2499 (48.9%) |
| **Age of children, Mean (SD)** | 0.156 (0.363) | 0.157 (0.363) | 0.156 (0.363) |
| **Region of Residence** |  |  |  |
| Major City | 1283 (70.4%) | 2098 (63.9%) | 3381 (66.2%) |
| Rest of state | 538 (29.5%) | 1176 (35.8%) | 1714 (33.6%) |
| Missing | 2 (0.1%) | 10 (0.3%) | 12 (0.2%) |
| **Caregiver’s characteristics** |  |  |  |
| **Age of caregivers** |  |  |  |
| Mean (SD) | 32.13 (4.87) | 30.74 (5.74) | 31.01 (5.51) |
| Missing, n | 0 (0%) | 1 (<0.01%) | 1 (<0.01%) |
| **Body mass index of caregivers** |  |  |  |
| Thinness/underweight | 164 (9.0%) | 310 (9.4%) | 474 (9.3%) |
| Normal | 783 (43.0%) | 1038 (31.6%) | 1821 (35.7%) |
| Overweight | 408 (22.4%) | 629 (19.2%) | 1037 (20.3%) |
| Obese | 257 (14.1%) | 470 (14.3%) | 727 (14.2%) |
| Missing | 211 (11.6%) | 837 (25.5%) | 1048 (20.5%) |
| **Partner of caregivers** |  |  |  |
| No | 89 (4.9%) | 394 (12.0%) | 483 (9.5%) |
| Yes | 1734 (95.1%) | 2890 (88.0%) | 4624 (90.5%) |
| **Sex of caregivers** |  |  |  |
| Male | 24 (1.3%) | 50 (1.5%) | 74 (1.4%) |
| Female | 1799 (98.7%) | 3234 (98.5%) | 5033 (98.6%) |

**Note:** Child covered by a health care card and SEIFA disadvantage quintile were not measured at Wave 1.

**Table S4.** Association of children’s disability status, disability types, and number of disabilities (wave 6) with caregivers’ health-related quality of life (Child Health CheckPoint data between wave 6 and 7).

|  |  | **Model 1: Utility score** | | **Model 2: PSD score** | | **Model 3: PsySD score** | |
| --- | --- | --- | --- | --- | --- | --- | --- |
|  | **Risk Factor** | **β (95% CI)** | **p-value** | **β (95% CI)** | **p-value** | **β (95% CI)** | **p-value** |
| Set 1 | **Disability** | |  |  |  |  |  |
|  | No | ref |  | **ref** |  | **ref** |  |
|  | Yes | -0.0334 (-0.0601, -0.0067) | 0.014 | -0.0278 (-0.0567, 0.0011) | 0.059 | -0.0338 (-0.0652, -0.0024) | 0.035 |
| Set 2 | **Types of disability** | |  |  |  |  |  |
|  | **Physical disability** | |  |  |  |  |  |
|  | No | ref |  | **ref** |  | **ref** |  |
|  | Yes | -0.0502 (-0.1217, 0.0214) | 0.169 | -0.0381 (-0.1157, 0.0394) | 0.334 | -0.0496 (-0.1340, 0.0346) | 0.247 |
|  | **Sensory disability** | |  |  |  |  |  |
|  | No | ref |  | **ref** |  | **ref** |  |
|  | Yes | -0.0386 (-0.0870, 0.0098) | 0.118 | -0.0382 (-0.0906, 0.0141) | 0.152 | -0.0365 (-0.0934, 0.0204) | 0.208 |
|  | **Psychosocial disability** | |  |  |  |  |  |
|  | No | ref |  | **ref** |  | **ref** |  |
|  | Yes | -0.0912 (-0.1637, -0.0185) | 0.014 | -0.0452 (-0.1238, 0.0334) | 0.260 | -0.0742 (-0.1596, 0.0113) | 0.089 |
|  | **Other disabilities/LTCs** | |  |  |  |  |  |
|  | No | ref |  | **ref** |  | **ref** |  |
|  | Yes | 0.0015 (-0.0322, 0.0352) | 0.931 | 0.0039 (-0.0404, 0.0326) | 0.834 | 0.0001 (-0.0396, 0.0398) | 0.994 |
| Set 3 | **Number of disabilities** | |  |  |  |  |  |
|  | No | ref |  | **ref** |  | **ref** |  |
|  | Single | -0.0274 (-0.0585, 0.0037) | 0.084 | -0.0194 (-0.0530, 0.0142) | 0.257 | -0.0340 (-0.0705, 0.0025) | 0.068 |
|  | Multiple | -0.0491 (-0.0986, 0.0005) | 0.052 | -0.0498 (-0.1034, 0.0037) | 0.068 | -0.0333 (-0.0915, 0.0249) | 0.262 |

***Note:*** Analysis of model in Set 1, 2 and 3 adjusted for sex of children, age of children, child covered by a health care card, region of residence, SEIFA disadvantage quintile, age of caregivers, body mass index of caregivers, partner of caregivers, and sex of caregivers; β, regression coefficient. ***Abbreviations:*** PSD, Physical super dimension; PsySD, Psychological super dimension; ref, reference category; CI, confidence interval.

**Figure**

| 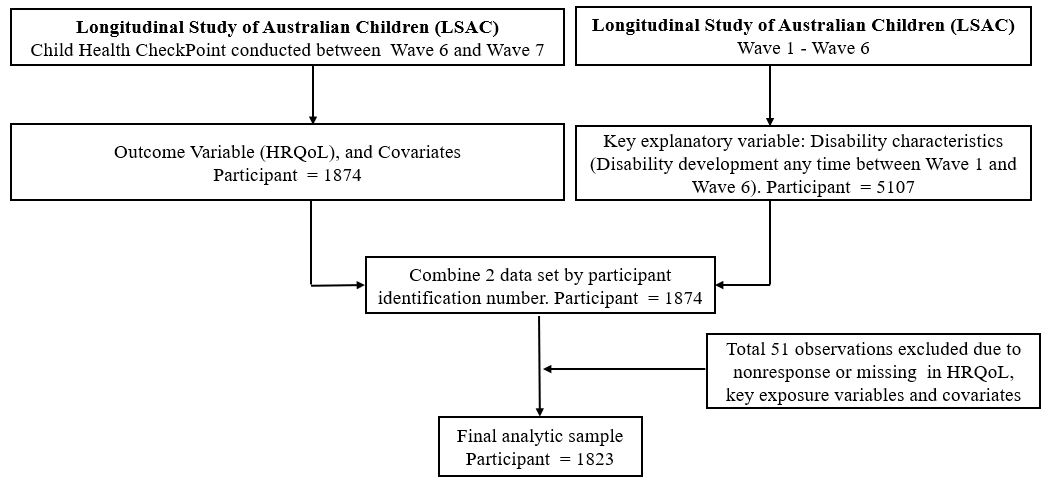 |
| --- |

**Figure S1.** Overview of participant inclusion, exclusion, and missing data patterns.
