# Supplementary material for: Children’s disability and caregivers’ health-related quality of life in Australia: A nationwide longitudinal study
Source: Eur J Pediatr. 2026 Jun 23;185(7):519. doi: 10.1007/s00431-026-07124-w (PMC13290929; doi:10.1007/s00431-026-07124-w)
Supplement: Supplementary file 2 — (PDF 222 KB) [file 431_2026_7124_MOESM2_ESM.pdf]

Children's disability and caregivers' health-related quality of life in Australia: a nationwide longitudinal study

Supplementary Material

Tables

Table S1. Definition of different types of disability.

Disability type	No.	Description	Response	Definition
**Physical**	1	Limited use of arms or fingers	No/Yes	A "yes" response to at least one of the six questions was classified as a physical disability; otherwise, it was not.
	2	Difficulty gripping things	No/Yes	
	3	Limited use of legs and feet	No/Yes	
	4	Any condition that restricts physical work (For example, back problems, migraines)	No/Yes	
	5	Physical disfigurement or deformity	No/Yes	
	6	Has chronic or recurring pain or discomfort, causing restriction	No/Yes	
**Sensory**	1	Has sight problems not corrected by glasses or contact lenses	No/Yes	A "yes" response to at least one of the three questions was classified as a sensory disability; otherwise, it was not.
	2	Has hearing problems	No/Yes	
	3	Has speech problems	No/Yes	
**Psychosocial**	1	Has a nervous or emotional condition causing restriction	No/Yes	A "yes" response to at least one of the two questions was classified as a psychosocial disability; otherwise, it was not.
	2	Has a mental illness for which help or supervision is required	No/Yes	
**Other disabilities/ long-term conditions (LTCs)**	1	Has shortness of breath or breathing difficulties, causing restriction	No/Yes	A "yes" response to at least one of the six questions was classified as an other disabilities/LTCs; otherwise, it was not.
	2	Has blackouts, fits or loss of consciousness	No/Yes	
	3	Has difficulty learning or understanding things	No/Yes	
	4	Has a long-term condition or ailment that is still restrictive even though it is being treated or medication is being taken for it	No/Yes	
	5	Has other long-term conditions such as arthritis, asthma, heart disease, Alzheimer's disease, dementia etc	No/Yes	
	6	Long-term effects as a result of a head injury, stroke or other brain damage	No/Yes	

Table S2. Summary of missing observations.

Variable	Total observation	Missing observation	Percent Missing
**Outcome variables**			
HRQoL, AOQL-9D utility score	1874	13	0.7%
PSD score	1874	11	0.6%
PsySD score	1874	13	0.7%
**Covariates**			
Sex of children	1874	0	0%
Age of children	1874	0	0%
Child covered by a health care card	1874	19	1.0%
Region of Residence	1874	0	0%
SEIFA disadvantage quintile	1874	0	0%
Age of caregivers	1874	0	0%
Sex of caregivers	1874	0	0%
Body mass index of caregivers	1874	14	0.7%
Partner of caregivers	1874	10	0.5%
**Key explanatory variable(s)**			
Disability status	1874	14	0.7%
Physical disability	1874	14	0.7%
Sensory disability	1874	14	0.7%
Psychosocial disability	1874	14	0.7%
Other disabilities/LTCs	1874	14	0.7%
Number of disabilities	1874	14	0.7%

Abbreviations: HRQoL, Health related Quality of Life ; PSD, Physical super dimension; PsySD, Psychological super dimension.

Table S3. Baseline characteristics of LSAC Wave 1 participants (n=5107), comparing included (n=1823) and excluded participants (n = 3284).

**Factors**	**Final analytic sample (N=1823)**	**Not included in the final analytic sample (N=3284)**	**Overall (N=5107)**
**Children characteristics**			
**Sex of children**			
Male	927 (50.9%)	1681 (51.2%)	2608 (51.1%)
Female	896 (49.1%)	1603 (48.8%)	2499 (48.9%)
**Age of children, Mean (SD)**	0.156 (0.363)	0.157 (0.363)	0.156 (0.363)
**Region of Residence**			
Major City	1283 (70.4%)	2098 (63.9%)	3381 (66.2%)
Rest of state	538 (29.5%)	1176 (35.8%)	1714 (33.6%)
Missing	2 (0.1%)	10 (0.3%)	12 (0.2%)
**Caregiver's characteristics**			
**Age of caregivers**			
Mean (SD)	32.13 (4.87)	30.74 (5.74)	31.01 (5.51)
Missing, n	0 (0%)	1 (<0.01%)	1 (<0.01%)
**Body mass index of caregivers**			
Thinness/underweight	164 (9.0%)	310 (9.4%)	474 (9.3%)
Normal	783 (43.0%)	1038 (31.6%)	1821 (35.7%)
Overweight	408 (22.4%)	629 (19.2%)	1037 (20.3%)
Obese	257 (14.1%)	470 (14.3%)	727 (14.2%)
Missing	211 (11.6%)	837 (25.5%)	1048 (20.5%)
**Partner of caregivers**			
No	89 (4.9%)	394 (12.0%)	483 (9.5%)
Yes	1734 (95.1%)	2890 (88.0%)	4624 (90.5%)
**Sex of caregivers**			
Male	24 (1.3%)	50 (1.5%)	74 (1.4%)
Female	1799 (98.7%)	3234 (98.5%)	5033 (98.6%)

Note: Child covered by a health care card and SEIFA disadvantage quintile were not measured at Wave 1.

Table S4. Association of children's disability status, disability types, and number of disabilities (wave 6) with caregivers' health-related quality of life (Child Health CheckPoint data between wave 6 and 7).

		**Model 1: Utility score**		**Model 2: PSD score**		**Model 3: PsySD score**	
	**Risk Factor**	**β (95% CI)**	**p-value**	**β (95% CI)**	**p-value**	**β (95% CI)**	**p-value**
Set 1	**Disability**						
	No	ref		**ref**		**ref**	
	Yes	-0.0334 (-0.0601, -0.0067)	0.014	-0.0278 (-0.0567, 0.0011)	0.059	-0.0338 (-0.0652, -0.0024)	0.035
Set 2	**Types of disability**						
	**Physical disability**						
	No	ref		**ref**		**ref**	
	Yes	-0.0502 (-0.1217, 0.0214)	0.169	-0.0381 (-0.1157, 0.0394)	0.334	-0.0496 (-0.1340, 0.0346)	0.247
	**Sensory disability**						
	No	ref		**ref**		**ref**	
	Yes	-0.0386 (-0.0870, 0.0098)	0.118	-0.0382 (-0.0906, 0.0141)	0.152	-0.0365 (-0.0934, 0.0204)	0.208
	**Psychosocial disability**						
	No	ref		**ref**		**ref**	
	Yes	-0.0912 (-0.1637, -0.0185)	0.014	-0.0452 (-0.1238, 0.0334)	0.260	-0.0742 (-0.1596, 0.0113)	0.089
	**Other disabilities/LTCs**						
	No	ref		**ref**		**ref**	
	Yes	0.0015 (-0.0322, 0.0352)	0.931	0.0039 (-0.0404, 0.0326)	0.834	0.0001 (-0.0396, 0.0398)	0.994
Set 3	**Number of disabilities**						
	No	ref		**ref**		**ref**	
	Single	-0.0274 (-0.0585, 0.0037)	0.084	-0.0194 (-0.0530, 0.0142)	0.257	-0.0340 (-0.0705, 0.0025)	0.068
	Multiple	-0.0491 (-0.0986, 0.0005)	0.052	-0.0498 (-0.1034, 0.0037)	0.068	-0.0333 (-0.0915, 0.0249)	0.262

Note: Analysis of model in Set 1, 2 and 3 adjusted for sex of children, age of children, child covered by a health care card, region of residence, SEIFA disadvantage quintile, age of caregivers, body mass index of caregivers, partner of caregivers, and sex of caregivers; β, regression coefficient. ***Abbreviations:*** PSD, Physical super dimension; PsySD, Psychological super dimension; ref, reference category; CI, confidence interval.

Figure

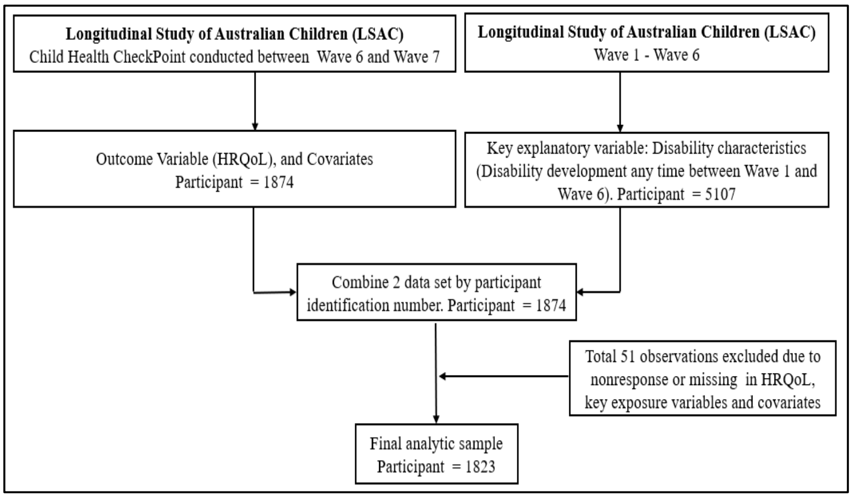

Figure S1. Overview of participant inclusion, exclusion, and missing data patterns.
